# US CDC Real-Time Reverse Transcription PCR Panel for Detection of Severe Acute Respiratory Syndrome Coronavirus 2

**DOI:** 10.3201/eid2608.201246

**Published:** 2020-08

**Authors:** Xiaoyan Lu, Lijuan Wang, Senthilkumar K. Sakthivel, Brett Whitaker, Janna Murray, Shifaq Kamili, Brian Lynch, Lakshmi Malapati, Stephen A. Burke, Jennifer Harcourt, Azaibi Tamin, Natalie J. Thornburg, Julie M. Villanueva, Stephen Lindstrom

**Affiliations:** Centers for Disease Control and Prevention, Atlanta, Georgia, USA (X. Lu, B. Whitaker, S.A. Burke, J. Harcourt, A. Tamin, N.J. Thornburg, J.M. Villanueva, S. Lindstrom);; Synergy America, Inc., Atlanta (L. Wang, S. Kamili); Eagle Contracting, Atlanta (S.K. Sakthivel, J. Murray, B. Lynch);; Leidos, Atlanta (L. Malapati); Battelle, Atlanta (S.A. Burke)

**Keywords:** 2019 novel coronavirus disease, coronavirus disease, COVID-19, severe acute respiratory syndrome coronavirus 2, SARS-CoV-2, viruses, respiratory infections, zoonoses, real-time reverse transcription PCR, real-time RT-PCR

## Abstract

Severe acute respiratory syndrome coronavirus 2 (SARS-CoV-2) was identified as the etiologic agent associated with coronavirus disease, which emerged in late 2019. In response, we developed a diagnostic panel consisting of 3 real-time reverse transcription PCR assays targeting the nucleocapsid gene and evaluated use of these assays for detecting SARS-CoV-2 infection. All assays demonstrated a linear dynamic range of 8 orders of magnitude and an analytical limit of detection of 5 copies/reaction of quantified RNA transcripts and 1 x 10^−1.5^ 50% tissue culture infectious dose/mL of cell-cultured SARS-CoV-2. All assays performed comparably with nasopharyngeal and oropharyngeal secretions, serum, and fecal specimens spiked with cultured virus. We obtained no false-positive amplifications with other human coronaviruses or common respiratory pathogens. Results from all 3 assays were highly correlated during clinical specimen testing. On February 4, 2020, the Food and Drug Administration issued an Emergency Use Authorization to enable emergency use of this panel.

On December 31, 2019, an outbreak of an unexplained acute respiratory disease, later designated coronavirus disease (COVID-19), was reported in Wuhan, China (*1*). On January 7, 2020, a novel coronavirus, severe acute respiratory syndrome coronavirus 2 (SARS-CoV-2), previously known as 2019-nCoV, was identified as the causative agent of the outbreak (*2*). On January 10, 2020, a SARS-CoV-2 genome sequence was shared with the global scientific community through an online resource (*3*). The virus was genetically most closely related to SARS-CoV and SARS-related bat and civet coronaviruses within the family *Betacoronavirus*, subgenus *Sarbecovirus* (*4*,*5*).

To support the potential public health emergency response to COVID-19, the Centers for Disease Control and Prevention (CDC) developed and validated a real-time reverse transcription PCR (rRT-PCR) panel based on this SARS-CoV-2 genome sequence (*3*). The panel targeted the nucleocapsid protein (N) gene of SARS-CoV-2. The rRT-PCR panel was validated under the Clinical Laboratory Improvement Amendments (https://www.cms.gov/Regulations-and-Guidance/Legislation/CLIA) for CDC use for diagnosis of SARS-CoV-2 from respiratory clinical specimens. On January 20, 2020, the CDC rRT-PCR panel confirmed an early case of COVID-19 in the United States (*6*). The US Food and Drug Administration issued an Emergency Use Authorization to enable emergency use of the CDC rRT-PCR panel as an in vitro diagnostic test for SARS-CoV-2 (https://www.fda.gov/news-events/press-announcements/fda-takes-significant-step-coronavirus-response-efforts-issues-emergency-use-authorization-first). From January 18 through February 27, as part of the COVID-19 response, CDC tested 2,923 specimens from 998 persons for SARS-CoV-2.

As of April 22, ≈2,400,000 confirmed COVID-19 cases and ≈169,000 associated deaths had been identified globally, including ≈770,000 cases and ≈37,000 deaths in the United States (*7*). We describe the design and validation of the CDC rRT-PCR panel and present comprehensive data on its performance with multiple specimen types and clinical specimens tested during the early CDC COVID-19 response.

## Materials and Methods

### SARS-CoV-2 Cultured Virus and Other Respiratory Pathogens

SARS-CoV-2 was isolated in Vero cells from a respiratory specimen from an early US COVID-19 patient (*8*). We measured infectious virus titer of virus stock by 50% tissue culture infectious dose (TCID_50_) (2.01 × 10^6^ TCID_50_/mL) and inactivated the virus by gamma irradiation. The inactivated stock virus was used as reference material for assay evaluation. Cell culture stocks or clinical specimens containing other respiratory viruses/bacteria were used to evaluate assay specificity. Ten nasopharyngeal swab samples that had been collected in 2018 (before COVID-19) and were negative for respiratory viruses were also available for assay specificity evaluation.

### Clinical Specimens

We used the rRT-PCR panel for SARS-CoV-2 to test 2,923 clinical specimens collected from January 18 through February 27, 2020, from persons under investigation from 43 states and territories in the United States. Specimens included 2,437 specimens collected from 998 persons suspected to be infected who either met the initial definition of a COVID-19 person under investigation (*9*), were a close contact with a laboratory-confirmed COVID-19 case-patient, or had been repatriated to the United States from Wuhan, China, or the Diamond Princess cruise ship from Japan; and 486 specimens that were serially collected over time from 28 persons with COVID-19 confirmed at CDC. Respiratory specimens (90.2%) included nasopharyngeal swab samples (n = 1,220) and oropharyngeal swab samples (n = 1,208) in viral transport medium, nasal swab/wash samples (n = 7), sputum (n = 197), bronchoalveolar lavage fluid (n = 2), lung tissues (n = 2), tracheal aspirate (n = 1), and bronchial wash samples (n = 1). Nonrespiratory specimens (9.8%) included serum (n = 156), urine (n = 72), stool (n = 54), cerebrospinal fluid (n = 2), and pericardial fluid (n = 1).

### Specimen Processing and Nucleic Acid Extraction

We extracted total nucleic acid from 120 μL of respiratory specimens by using the EZ1 DSP Virus Kit (QIAGEN, https://www.qiagen.com) following the manufacturer’s instructions and collected 120 μL elution volumes. We processed sputum specimens by adding equal volumes of 10 mM of freshly prepared No-Weigh Thermo Scientific Pierce dithiothreitol (Thermo Fisher Scientific, https://www.thermofisher.com) and incubating them at room temperature for 30 min with intermittent mixing or until the specimens were sufficiently liquefied for extraction. We processed stool specimens by preparing 10% suspensions by adding 100 μL of liquid stool or ≈100 mg of solid stool to 900 μL of phosphate-buffered saline, pH 7.4 (Thermo Fisher Scientific), pulse vortexing for 30 s, and centrifuging at 4,000 × *g* for 10 min at 4°C. We then carefully removed the clarified supernatant for extraction. To demonstrate successful nucleic acid recovery and reagent integrity, we extracted human specimen control consisting of cultured A549 cells concurrently with the test specimens as a procedural control. We either tested extracts immediately or stored them at <−70°C until use.

### Primers and Probes

We aligned the N gene sequence from the publicly available SARS-CoV-2 genome (GenBank accession no. MN908947) with other coronavirus sequences available from GenBank by using MAFFT version 7.450 implemented in Geneious Prime (Geneious Biologics, https://www.geneious.com). We designed multiple primer/probe sets targeting regions in the 5′, middle, and 3′ regions of the N gene sequence with the aid of Primer Express software version 3.0.1 (Thermo Fisher Scientific). We selected 3 candidate gene regions, designated N1, N2, and N3, for further study (Table 1). N1 and N2 were designed to specifically detect SARS-CoV-2, and N3 was designed to universally detect all currently recognized clade 2 and 3 viruses within the subgenus *Sarbecovirus* (*4*), including SARS-CoV-2, SARS-CoV, and bat- and civet-SARS–like CoVs. BLASTn (http://www.ncbi.nlm.nih.gov/blast/Blast.cgi) analysis demonstrated no major combined similarity of primers and probes of each assay with other coronaviruses (OC43, 229E, HKU1, NL63, and Middle East respiratory syndrome coronavirus [MERS-CoV]) or microflora of humans that would potentially yield false-positive results. We synthesized all primers and probes by using standard phosphoramidite chemistry techniques at the CDC Biotechnology Core Facility. We labeled hydrolysis probes at the 5′ end with 6-carboxy-fluorescein (FAM) and at the 3′ end with Black Hole Quencher 1 (Biosearch Technologies, https://www.biosearchtech.com).

**Table 1:** Assay primer/probe sequences for the US CDC rRT-PCR panel for detection of SARS-CoV-2*

Assay	Genome target	Genome location	Primers and probes†	Sequence, 5′→3′	Amplicon size, bp	Assay use
N1	Nucleocapsid gene	28303–28322‡	Forward primer	GACCCCAAAATCAGCGAAAT	73	SARS-CoV-2
		28374–28351‡	Reverse primer	TCTGGTTACTGCCAGTTGAATCTG		
		28325–28348‡	Probe	ACCCCGCATTACGTTTGGTGGACC		
N2	Nucleocapsid gene	29180–29199‡	Forward primer	TTACAAACATTGGCCGCAAA	67	SARS-CoV-2
		29246–29228‡	Reverse primer	GCGCGACATTCCGAAGAA		
		29204–29226‡	Probe	ACAATTTGCCCCCAGCGCTTCAG		
N3	Nucleocapsid gene	28697–28718‡	Forward primer	GGGAGCCTTGAATACACCAAAA	72	SARS-CoV-2, SARS-CoV, and other *Sarbecoviruses*§
		28768–28748‡	Reverse primer	TGTAGCACGATTGCAGCATTG		
		28720–28743‡	Probe	AYCACATTGGCACCCGCAATCCTG		
RP¶	Human RNase P gene	50–68#	Forward primer	AGATTTGGACCTGCGAGCG	65	Sample quality control
		114–95#	Reverse primer	GAGCGGCTGTCTCCACAAGT		
		71–93#	Probe	TTCTGACCTGAAGGCTCTGCGCG		

*CDC, Centers for Disease Control and Prevention; N, nucleocapsid protein gene; RP, ribonuclease P gene; rRT-PCR, real-time reverse transcription PCR; SARS-CoV-2, severe acute respiratory coronavirus 2. †Probes labeled at the 5′-end with the reporter molecule 6-carboxyfluorescein (FAM) and at the 3′-end with Black Hole Quencher 1 (Biosearch Technologies Inc., https://www.biosearchtech.com). ‡Nucleotide numbering based on SARS-CoV-2 (accession no. MN908947). §Bat- and civet-SARS–like coronaviruses. ¶Primer/probe sequences from (*10*).  #Nucleotide numbering based on human RP mRNA (accession no. NM_006413).

### In Vitro RNA Transcript and Viral Template Control

Double-stranded DNA containing a 5′-T7 RNA polymerase promoter sequence (TAATACGACTCACTATAGGG) and the SARS-CoV-2 complete N gene sequence was synthesized by Integrated DNA Technologies (https://www.idtdna.com**)**. We transcribed the DNA by using the MEGAscript T7 Transcription Kit (Thermo Fisher Scientific). The RNA transcripts were purified by using the MEGAclear Transcription Clean-Up Kit (Thermo Fisher Scientific) and quantified with a Qubit fluorometer by using a Qubit RNA HS Assay Kit (Thermo Fisher Scientific). We used the RNA transcript for subsequent assay evaluation and positive template control.

### rRT-PCR Assay

We performed all rRT-PCR testing by using the TaqPath 1-Step RT-qPCR Master Mix, CG (Thermo Fisher Scientific). Each 20-μL reaction contained 5 μL of 4X Master Mix (Thermo Fisher Scientific), 0.5 μL of 5 μmol/L probe, 0.5 μL each of 20 μmol/L forward and reverse primers, 8.5 μL of nuclease-free water, and 5 μL of nucleic acid extract. We conducted amplification in 96-well plates on an Applied Biosystems 7500 Fast Dx Real-Time PCR Instrument (Thermo Fisher Scientific). Thermocycling conditions consisted of 2 min at 25°C for uracil-DNA glycosylase incubation, 15 min at 50°C for reverse transcription, 2 min at 95°C for activation of the Taq enzyme, and 45 cycles of 3 s at 95°C and 30 s at 55°C. We set the threshold in the middle of exponential amplification phase in log view. A positive test result was defined as an exponential fluorescent curve that crossed the threshold within 40 cycles (cycle threshold [C_t_] <40).

### Test Algorithm

For routine specimen testing, we ran all 3 SARS-CoV-2 assays simultaneously along with the human ribonuclease P gene (RP) assay to monitor nucleic acid extraction, specimen quality, and presence of reaction inhibitors. To monitor assay performance, we included positive template controls, no-template controls, and human specimen controls in all runs. When all controls exhibited expected performance, we considered a specimen to be positive for SARS-CoV-2 if all assay amplification curves crossed the threshold line within 40 cycles (C_t_ <40). If all 3 assay results were negative, the test result was reported as negative. If all 3 assay results were not positive, an inconclusive test result was recorded and retesting was required. If both initial and retest results were inconclusive, the final result was reported as inconclusive.

## Results

### Assay Efficiency

**Figure 1:**
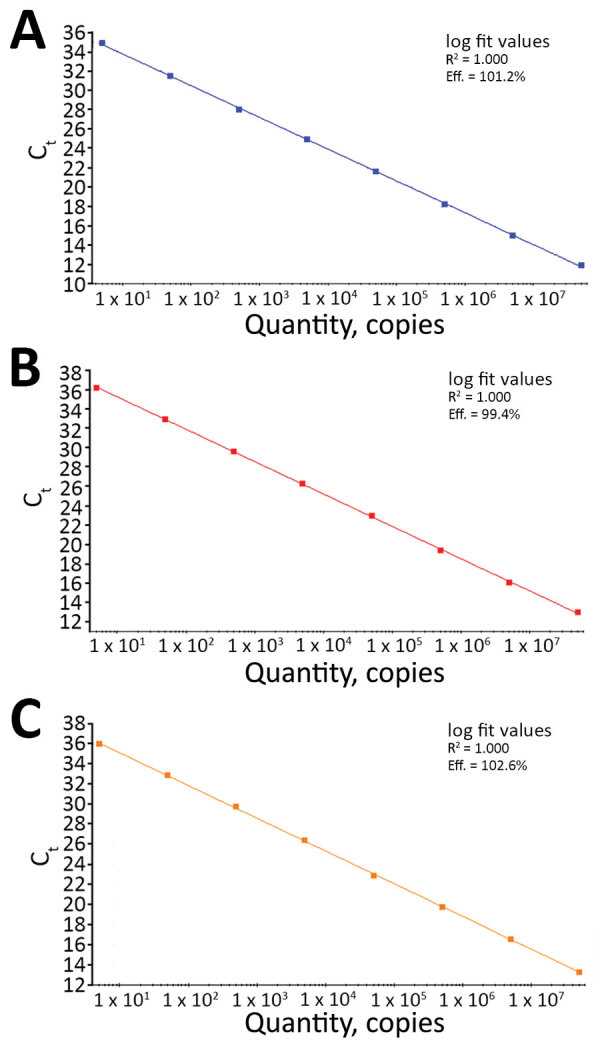
Linear regression analysis of serial 10-fold dilutions of synthetic RNA transcripts of the nucleocapsid gene (N) ranging from 5 to 5 × 10^7^ copies/reaction tested by the N1 (A), N2 (B), and N3 (C) assays in the US Centers for Disease Control and Prevention real-time reverse transcription PCR panel for detection of severe acute respiratory syndrome coronavirus 2. For each assay, R^2^ indicates calculated linear correlation coefficients and eff. indicates amplification efficiencies. C_t_, cycle threshold.

We prepared serial 10-fold dilutions of quantified RNA transcript in 10 mmol/L Tris-HCl buffer containing 50 ng/µL of yeast tRNA (Thermo Fisher Scientific) and tested them by each assay. A linear amplification was achieved over an 8-log dynamic range from 5 to 5 × 10^7^ copies/reaction for all 3 assays; calculated efficiency ranged from 99.4% to 102.6% (Figure 1).

### Analytical Sensitivity (Limits of Detection)

#### SARS-CoV-2 RNA Transcripts

 We tested serial 2-fold dilutions of quantified RNA transcript prepared in buffer as above by each assay in 24 replicates/dilution. The highest dilution of transcript at which all replicates were positive was defined as the limit of detection (LoD) for each assay. The LoDs for all assays were 5 RNA transcript copies/reaction (Table 2), which is consistent with that previously demonstrated (*11*).

**Table 2:** Limits of detection of the US CDC rRT-PCR panel for detection of SARS-CoV-2 with RNA transcripts*

Copies/reaction	No. positive tests/no. (%) reaction replicates		
	N1	N2	N3
20	24/24 (100)	24/24 (100)	24/24 (100)
10	24/24 (100)	24/24 (100)	24/24 (100)
5	24/24 (100)	24/24 (100)	24/24 (100)
2.5	23/24 (95.8)	16/24 (66.7)	15/24 (62.5)
1.25	15/24 (62.5)	8/24 (33.3)	3/24 (12.5)

*CDC, Centers for Disease Control and Prevention; N, nucleocapsid protein gene; rRT-PCR, real-time reverse transcription PCR; SARS-CoV-2, severe acute respiratory coronavirus 2.

#### SARS-CoV-2 Genomic RNA

We tested serial half-log dilutions of SARS-CoV-2 RNA extracted from inactivated cultured virus and prepared in buffer as above in triplicate by each assay (Table 3). For all 3 assays, the LoD was ≈1 × 10^−1.5^ TCID_50_/mL. 

**Table 3:** Limits of detection of the US CDC rRT-PCR panel for detection of SARS-CoV-2 with extracted SARS-CoV-2 virus RNA*

Virus concentration, TCID_50_	N1 C_t_					N2 C_t_					N3 C_t_			
	Test 1	Test 2	Test 3	Call rate		Test 1	Test 2	Test 3	Call rate		Test 1	Test 2	Test 3	Call rate
1 × 10^0.5^	27.5	27.1	27.4	3/3		29.2	28.9	28.7	3/3		28.3	28.3	28.2	3/3
1 × 10^0^	30.9	29.4	29.9	3/3		31.2	31.1	31.1	3/3		30.0	30.0	30.0	3/3
1 × 10^−0.5^	30.7	30.9	31.1	3/3		33.0	32.7	32.3	3/3		31.4	31.4	32.5	3/3
1 × 10^−1^	33.0	32.4	32.9	3/3		34.4	34.3	34.7	3/3		34.6	32.3	33.3	3/3
1 × 10^−1.5^	34.4	33.6	35.6	3/3		36.3	36.1	37.6	3/3		35.4	35.8	35.6	3/3
1 × 10^−2^	37.2	36.1	Neg	2/3		39.0	Neg	Neg	1/3		36.1	Neg	Neg	1/3
1 × 10^-2.5^	36.2	36.3	Neg	2/3		38.8	37.6	Neg	2/3		37.1	Neg	Neg	1/3
1 × 10^−3^	Neg	Neg	Neg	0/3		Neg	Neg	Neg	0/3		Neg	Neg	Neg	0/3

*Call rate indicates each assay performed in triplicate. CDC, Centers for Disease Control and Prevention; C_t_, cycle threshold; N, nucleocapsid protein gene; neg, negative; rRT-PCR, real-time reverse transcription PCR; SARS-CoV-2, severe acute respiratory coronavirus 2; TCID_50_, 50% tissue culture infectious dose.

#### SARS-CoV-2 Spiked in Different Clinical Matrices

Serial half-log dilutions of SARS-CoV-2 were spiked in different specimen matrices contrived from pooled human clinical specimens: 10 serum samples, 10 nasopharyngeal swab samples, 10 oropharyngeal swab samples, 10 sputum samples, and 10 stool suspensions (prepared as 10% suspensions). The LoDs for all assays ranged from 1.0 × 10^−1.5^ to 1.0 × 10^−1^ TCID_50_/mL across all specimen matrices (Table 4). Negative results were obtained for all 3 assays with all pooled negative specimen matrices.

**Table 4:** Performance of the US CDC rRT-PCR panel for detection of SARS-CoV-2 with various specimen matrices spiked with SARS-CoV-2*

Virus titer, TCID_50_†	N1 C_t_					N2 C_t_					N3 C_t_					RP C_t_			
	Test 1	Test 2	Test 3	Call rate		Test 1	Test 2	Test 3	Call rate		Test 1	Test 2	Test 3	Call rate		Test 1	Test 2	Test 3	Call rate
1 × 10^0.5^																			
NP	30.4	29.9	29.9	3/3		30.3	30.5	30.5	3/3		30.0	29.6	29.7	3/3		26.0	26.1	26.1	3/3
OP	30.1	29.7	29.7	3/3		30.7	30.8	30.5	3/3		29.6	29.5	29.5	3/3		30.0	30.3	30.2	3/3
Sputum	30.3	30.1	30.4	3/3		31.2	31.5	31.7	3/3		30.5	30.0	30.4	3/3		24.9	24.8	25.1	3/3
Serum	30.1	29.7	29.8	3/3		31.1	31.0	31.1	3/3		29.8	29.8	29.6	3/3		29.1	28.6	28.3	3/3
Stool	30.7	30.5	30.7	3/3		31.7	31.9	31.7	3/3		30.7	29.9	30.2	3/3		34.9	35.1	35.7	3/3
1 × 10^0^																			
NP	32.1	30.8	30.4	3/3		32.3	32.0	31.6	3/3		31.3	30.7	30.9	3/3		24.5	24.2	25.0	3/3
OP	31.6	31.3	31.4	3/3		32.8	32.3	32.2	3/3		30.8	31.3	31.1	3/3		29.3	29.2	29.5	3/3
Sputum	32.0	32.0	31.8	3/3		33.1	32.9	32.7	3/3		31.7	31.4	32.1	3/3		24.3	24.3	24.6	3/3
Serum	32.2	30.8	31.4	3/3		32.4	32.6	33.1	3/3		31.2	31.3	31.3	3/3		28.1	28.2	27.5	3/3
Stool	32.1	32.3	32.0	3/3		33.6	33.9	33.5	3/3		32.1	32.0	32.1	3/3		34.6	35.1	34.5	3/3
1 × 10^–0.5^																			
NP	33.7	32.5	33.9	3/3		34.1	33.9	35.5	3/3		33.2	32.6	33.5	3/3		25.3	25.4	25.5	3/3
OP	33.6	33.6	33.1	3/3		34.4	34.4	34.6	3/3		33.5	33.0	32.0	3/3		29.2	29.4	29.6	3/3
Sputum	35.1	33.4	33.0	3/3		35.0	34.2	34.8	3/3		33.5	33.4	33.2	3/3		24.0	24.2	24.3	3/3
Serum	33.4	32.2	33.3	3/3		35.2	34.1	33.9	3/3		32.7	32.7	33.1	3/3		28.3	28.2	29.3	3/3
Stool	35.0	35.3	35.3	3/3		36.2	36.4	35.3	3/3		34.2	34.6	34.0	3/3		33.4	36.2	35.0	3/3
1 × 10^–1^																			
NP	33.9	34.0	34.6	3/3		36.0	36.2	36.5	3/3		34.1	34.3	35.1	3/3		25.6	25.6	25.9	3/3
OP	33.4	33.3	33.6	3/3		35.9	36.7	35.3	3/3		34.5	34.3	35.1	3/3		29.2	29.3	29.3	3/3
Sputum	35.2	35.0	36.2	3/3		36.8	36.8	35.3	3/3		35.3	35.2	35.1	3/3		24.1	24.1	24.3	3/3
Serum	37.5	35.3	34.8	3/3		36.4	37.2	36.3	3/3		35.2	33.7	34.3	3/3		28.3	28.3	28.6	3/3
Stool	36.1	35.8	36.0	3/3		37.3	37.9	38.1	3/3		35.8	35.6	34.7	3/3		34.1	33.9	34.2	3/3
1 × 10^–1.5^																			
NP	35.6	36.8	35.9	3/3		39.9	36.8	37.6	3/3		36.7	35.1	35.7	3/3		26.1	26.3	26.6	3/3
OP	36.2	35.2	Neg	2/3		36.8	38.0	Neg	2/3		Neg	Neg	Neg	0/3		29.3	29.3	29.6	3/3
Sputum	36.9	36.3	Neg	2/3		39.1	39.5	38.4	3/3		35.8	38.2	Neg	2/3		23.9	24.2	24.2	3/3
Serum	36.8	36.5	36.4	3/3		38.4	39.1	36.9	3/3		35.6	36.2	Neg	2/3		27.9	28.2	27.4	3/3
Stool	Neg	Neg	Neg	0/3		39.2	38.1	37.5	3/3		35.5	38.1	38.0	3/3		34.2	33.3	35.6	3/3
1 × 10^–2^																			
NP	Neg	Neg	Neg	0/3		39.4	Neg	Neg	1/3		Neg	Neg	Neg	0/3		25.5	25.7	25.8	3/3
OP	Neg	Neg	Neg	0/3		38.5	38.0	Neg	2/3		37.1	Neg	Neg	1/3		29.4	29.3	29.4	3/3
Sputum	36.1	Neg	Neg	1/3		38.2	38.5	Neg	2/3		37.1	37.5	Neg	2/3		24.0	24.0	24.1	3/3
Serum	37.5	Neg	Neg	1/3		39.9	Neg	Neg	1/3		38.9	Neg	Neg	1/3		28.2	27.9	27.2	3/3
Stool	Neg	Neg	Neg	0/3		39.1	Neg	Neg	1/3		38.1	Neg	Neg	1/3		33.5	35.1	34.6	3/3
1 × 10^–2.5^																			
NP	36.2	Neg	Neg	1/3		38.9	Neg	Neg	1/3		Neg	Neg	Neg	0/3		26.3	26.5	26.6	3/3
OP	Neg	Neg	Neg	0/3		Neg	Neg	Neg	0/3		Neg	Neg	Neg	0/3		29.0	29.2	29.2	3/3
Sputum	37.4	Neg	Neg	1/3		Neg	Neg	Neg	0/3		36.7	Neg	Neg	1/3		24.0	24.2	24.4	3/3
Serum	36.4	Neg	Neg	1/3		38.4	Neg	Neg	1/3		Neg	Neg	Neg	0/3		28.1	28.2	27.2	3/3
Stool	37.6	Neg	Neg	1/3		Neg	Neg	Neg	0/3		Neg	Neg	Neg	0/3		33.4	34.1	35.5	3/3
1 × 10^–3^																			
NP	Neg	Neg	Neg	0/3		Neg	Neg	Neg	0/3		Neg	Neg	Neg	0/3		26.8	26.7	27.0	3/3
OP	Neg	Neg	Neg	0/3		Neg	Neg	Neg	0/3		Neg	Neg	Neg	0/3		29.2	29.4	29.1	3/3
Sputum	Neg	Neg	Neg	0/3		Neg	Neg	Neg	0/3		Neg	Neg	Neg	0/3		23.9	24.1	24.3	3/3
Serum	Neg	Neg	Neg	0/3		Neg	Neg	Neg	0/3		Neg	Neg	Neg	0/3		28.2	28.3	27.9	3/3
Stool	Neg	Neg	Neg	0/3		Neg	Neg	Neg	0/3		Neg	Neg	Neg	0/3		34.1	35.0	35.0	3/3
0																			
NP	Neg	Neg	Neg	0/3		Neg	Neg	Neg	0/3		Neg	Neg	Neg	0/3		25.0	25.2	24.8	3/3
OP	Neg	Neg	Neg	0/3		Neg	Neg	Neg	0/3		Neg	Neg	Neg	0/3		28.6	28.3	28.5	3/3
Sputum	Neg	Neg	Neg	0/3		Neg	Neg	Neg	0/3		Neg	Neg	Neg	0/3		23.1	23.0	23.1	3/3
Serum	Neg	Neg	Neg	0/3		Neg	Neg	Neg	0/3		Neg	Neg	Neg	0/3		27.6	27.8	27.7	3/3
Stool	Neg	Neg	Neg	0/3		Neg	Neg	Neg	0/3		Neg	Neg	Neg	0/3		33.5	33.8	33.2	3/3

*Call rate indicates each assay performed in triplicate. CDC, Centers for Disease Control and Prevention; C_t_, cycle threshold; N, nucleocapsid protein gene; neg, negative; NP, nasopharyngeal; OP, oropharyngeal; RP, ribonuclease P gene; rRT-PCR, real-time reverse transcription PCR; SARS-CoV-2, severe acute respiratory coronavirus 2; TCID_50_, 50% tissue culture infectious dose. †50% tissue culture infectious dose/mL.

### Assay Reproducibility

We evaluated assay reproducibility by using 3 contrived respiratory specimens constructed from pooled nasopharyngeal swabs and spiked with high (1.0 × 10^3^ TCID_50_/mL), moderate (1.0 × 10^1^ TCID_50_/mL), and low (1.0 × 10^−1^ TCID_50_/mL) concentrations of virus. We extracted the contrived specimens, and tested them in triplicate against each assay on 3 different days. Interassay variation was low for all assays (coefficient of variation range for N1, 1.8%–3.7%; N2, 2.3%–2.8%; N3, 1.1%–1.3%; RP, 0.9%–1.5%) (Table 5).

**Table 5:** Reproducibility of the US CDC rRT-PCR panel for detection of SARS-CoV-2 with respiratory specimen matrix spiked with SARS-CoV-2*

Virus titer, TCID_50_	N1 C_t_				N2 C_t_				N3 C_t_				RP C_t_		
	Test 1	Test 2	Test 3		Test 1	Test 2	Test 3		Test 1	Test 2	Test 3		Test 1	Test 2	Test 3
Day 1															
1.0 × 10^3^	21.1	21.1	21.0		21.5	21.2	21.5		21.0	21.1	21.1		26.0	25.9	26.1
1.0 × 10^1^	27.8	27.6	27.5		28.6	28.6	28.9		27.6	27.3	27.7		26.0	25.8	26.1
1.0 × 10^−1^	35.6	33.8	33.8		34.7	34.2	34.5		34.0	34.5	33.7		26.4	26.4	26.4
Day 2															
1.0 × 10^3^	21.8	21.8	21.8		21.6	21.5	21.6		21.2	21.1	21.2		26.5	26.3	26.3
1.0 × 10^1^	28.4	28.3	28.5		29.4	29.3	29.0		28.1	28.0	28.1		26.7	26.7	26.67
1.0 × 10^−1^	37.6	35.7	34.0		36.0	34.7	34.9		34.1	33.8	34.5		26.8	26.3	26.2
Day 3															
1.0 × 10^3^	20.8	20.7	20.8		20.6	20.4	20.6		20.7	20.6	20.7		26.8	26.7	26.9
1.0 × 10^1^	27.1	27.6	27.3		27.5	27.6	27.4		27.2	27.3	27.3		26.6	26.8	26.8
1.0 × 10^−1^	34.2	33.9	34.1		33.1	33.5	34.9		33.2	34.2	33.5		26.6	26.9	26.7
Summary results	Mean	SD	CV		Mean	SD	CV		Mean	SD	CV		Mean	SD	CV
1.0 × 10^3^	21.2	0.5	2.2%		21.2	0.5	2.3%		21.0	0.2	1.1%		26.4	0.4	1.4%
1.0 × 10^1^	27.8	0.5	1.8%		28.5	0.8	2.8%		27.6	0.4	1.3%		26.5	0.4	1.5%
1.0 × 10^−1^	34.7	1.3	3.7%		34.5	0.8	2.5%		33.9	0.4	1.2%		26.5	0.2	0.9%

*Specimen matrix constructed from combined nasopharyngeal swabs obtained from 10 persons. CDC, Centers for Disease Control and Prevention; C_t_, cycle threshold; neg, negative; CV, coefficient of variation; N, nucleocapsid protein gene; RP, ribonuclease P gene; rRT-PCR, real-time reverse transcription PCR; SARS-CoV-2, severe acute respiratory coronavirus 2; TCID_50_, 50% tissue culture infectious dose.  †50% tissue culture infectious dose/mL.

### Analytical Specificity

#### In Silico Analysis Against Available SARS-CoV-2 Genome Sequences

We evaluated the primer/probe sequences of the 3 assays against 7,158 genome sequences available from the Global Initiative on Sharing All Influenza Data (GISAID, https://www.gisaid.org) as of April 14, 2020. Nucleotide mismatches in the primer/probe regions with frequency >0.1% were sporadic among viruses (Table 6). Except for 1 nucleotide mismatch with frequency >1% (1.55%) at the eighth position of the N3 forward primer, frequency of all other mismatches was <1%. These nucleotide mismatches would not be expected to affect reaction performance. No viruses were found to have >1 mismatch in any primer/probe region.

**Table 6:** Nucleotide mismatches among 7,158 SARS-CoV-2 genome sequences found in the primer and probe regions included in the US CDC rRT-PCR panel for detection of SARS-CoV-2*

Primer/probe	N1 probe	N1 reverse		N2 forward	N3 forward		N3 probe		N3 reverse
Location, 5′→3′	3	15	21	4	8	10	5	17	14
Mismatch nucleotide	C>T	G>T	T>C	C>T	T>C	G>T	C>T	C>T	C>A
No. sequences	39	22	33	7	111	7	7	9	22
Mismatch frequency, %†	0.54	0.31	0.46	0.10	1.55	0.10	0.10	0.13	0.31

*CDC, Centers for Disease Control and Prevention; N, nucleocapsid protein gene; rRT-PCR, real-time reverse transcription PCR; SARS-CoV-2, severe acute respiratory coronavirus 2. †Only mismatches with frequency >0.10% are shown.

#### Cross-Reactivity with Other Respiratory Pathogens and Human Microbial Flora

We evaluated the specificity of the SARS-CoV-2 rRT-PCR assay with purified nucleic acid obtained from a collection of respiratory pathogen isolates or positive clinical specimens, including human coronaviruses 229E, OC43, NL63, HKU1, SARS-CoV, and MERS-CoV (Table 7). We also tested 10 nasopharyngeal swabs samples collected in 2018 before COVID-19 was identified. Except for the N3 assay, which was reactive with SARS-CoV RNA as expected, we observed no false-positive results for any pathogens and specimens tested.

**Table 7:** Cross-reactivity of the US CDC rRT-PCR panel for detection of SARS-CoV-2 against other respiratory pathogens*

Pathogen (strain)	Source	Other respiratory pathogens, rRT-PCR (C_t_)	SARS-CoV-2 rRT-PCR		
			N1	N2	N3 (C_t_)
Adenovirus C1 (Ad.71)	Virus isolate	Pos (14.0)	Neg	Neg	Neg
Bocavirus	Clinical specimen	Pos (14.9)	Neg	Neg	Neg
Coronavirus 229E	Virus isolate	Pos (9.6)	Neg	Neg	Neg
Coronavirus OC43	Virus isolate	Pos (12.9)	Neg	Neg	Neg
Coronavirus HKU1	Clinical specimen	Pos (22.3)	Neg	Neg	Neg
Coronavirus MERS	Virus isolate	Pos (14.3)	Neg	Neg	Neg
Coronavirus NL63	Clinical specimen	Pos (21.9)	Neg	Neg	Neg
Coronavirus SARS (Urbani)	Virus isolate	Pos (27.3)	Neg	Neg	Pos (26.3)†
Enterovirus D68	Virus isolate	Pos (21.3)	Neg	Neg	Neg
Human metapneumovirus (CAN 99–81)	Virus isolate	Pos (13.8)	Neg	Neg	Neg
Influenza A H1N1 (A/India/2012)	Virus isolate	Pos (14.7)	Neg	Neg	Neg
Influenza A H3N1 (A/Texas/2012)	Virus isolate	Pos (10.7)	Neg	Neg	Neg
Influenza B (B/Massachusetts/1999)	Virus isolate	Pos (8.4)	Neg	Neg	Neg
Parainfluenza 1 (C35)	Virus isolate	Pos (17.2)	Neg	Neg	Neg
Parainfluenza 2 (Greer)	Virus isolate	Pos (17.1)	Neg	Neg	Neg
Parainfluenza 3 (C-43)	Virus isolate	Pos (20.4)	Neg	Neg	Neg
Parainfluenza 4a (M-25)	Virus isolate	Pos (16.7)	Neg	Neg	Neg
Parainfluenza 4b (CH 19503)	Virus isolate	Pos (18.2)	Neg	Neg	Neg
Respiratory syncytial virus (Long)	Virus isolate	Pos (15.1)	Neg	Neg	Neg
Rhinovirus 1A	Virus isolate	Pos (15.9)	Neg	Neg	Neg
*Mycoplasma pneumoniae*	Cultured bacteria	Pos (20.7)	Neg	Neg	Neg
*Streptococcus pneumoniae*	Cultured bacteria	Pos (21.1)	Neg	Neg	Neg

*CDC, Centers for Disease Control and Prevention; C_t_, cycle threshold; neg, negative;pos, positive; MERS, Middle East respiratory syndrome; N, nucleocapsid protein gene; rRT-PCR, real-time reverse transcription PCR; SARS-CoV-2, severe acute respiratory coronavirus 2. †N3 assay designed for universal detection of clade 2 and 3 within *Sarbecovirus* subgenus including SARS-CoV-2, SARS-CoV, and bat- and civet-SARS–like coronaviruses.

### Clinical Specimen Testing

#### Specimens from Persons with Suspected Cases

Among the 2,437 clinical specimens collected from 998 persons with suspected cases for initial SARS-CoV-2 diagnostic testing, 81 (3.32%) specimens (42 nasopharyngeal, 33 oropharyngeal, 5 sputum, 1 BAL) collected from 46 persons with suspected cases were positive and 2,355 (96.64%) specimens were negative (Table 8). We did not detect SARS-CoV-2 RNA in any of the 74 serum and 10 urine specimens tested.

**Table 8:** Test results for 2,923 human specimens determined by the US CDC real-time RT-PCR panel for detection of SARS-CoV-2*

Specimens	Specimens for initial laboratory diagnosis, no. (%)					Serial follow-up specimens from laboratory- confirmed positive cases, no. (%)			
	Positive	Negative	Inconclusive	Total		Positive	Negative	Inconclusive	Total
Upper respiratory tract									
NP swab	42 (3.85)	1,048 (96.06)	1 (0.09)	1,091 (100)		60 (46.51)	50 (38.76)	19 (14.73)	129 (100)
OP swab	33 (3.09)	1,035 (96.91)	0	1,068 (100)		42 (30.00)	86 (61.43)	12 (8.57)	140 (100)
Nasal swab/wash	0	7 (100)	0	7 (100)		0	0	0	0
Lower respiratory tract									
Sputum	5 (2.79)	174 (97.21)	0	179 (100)		13 (72.22)	3 (16.67)	2 (11.11)	18 (100)
BAL	1 (50)	1 (50)	0	2 (100)		0	0	0	0
Bronchial wash	0	1 (100)	0	1 (100)		0	0	0	0
Tissue, lung	0	2 (100)	0	2 (100)		0	0	0	0
Tracheal aspirate	0	0	0	0		1 (100)	0	0	1 (100)
Other									
Serum	0	74 (100)	0	74 (100)		4 (4.88)	76 (92.68)	2 (2.44)	82 (100)
Stool	0	0	0	0		22 (40.74)	28 (51.85)	4 (7.41)	54 (100)
Urine	0	10 (100)	0	10 (100)		0	62 (100)	0	62 (100)
Pleural fluid	0	1 (100)	0	1 (100)		0	0	0	0
CSF	0	2 (100)	0	2 (100)		0	0	0	0
Total	81 (3.32)	2,355 (96.64)	1 (0.04)	2,437 (100)		142 (29.22)	305 (62.76)	39 (8.02)	486 (100)

*BAL, bronchoalveolar lavage; CDC, Centers for Disease Control and Prevention; CSF, cerebrospinal fluid; NP, nasopharyngeal; OP, oropharyngeal; rRT-PCR, real-time reverse transcription PCR; SARS-CoV-2, severe acute respiratory coronavirus 2.

#### Serially Collected Specimens from Persons with Laboratory-Confirmed COVID-19

Of 486 specimens serially collected from 28 persons with laboratory-confirmed COVID-19, results were SARS-CoV-2 positive for 142 (29.22%) samples (60 nasopharyngeal, 42 oropharyngeal, 13 sputum, 1 tracheal aspirate, 22 stool, and 4 serum) (Table 8). We detected SARS-CoV-2 RNA in serum of 2 of 15 persons with laboratory-confirmed COVID-19 for whom serum was available for testing. For 1 of those case-patients, serum was collected 14 days after symptom onset and tested positive. For the other case-patient, a total of 10 serum samples were collected. Of those, specimens collected on days 9, 11, and 13 were positive; specimens collected on days 3, 19, 22, 25, and 28 were negative; and specimens collected on days 6 and 16 had inconclusive results. A total of 22 stool specimens collected from 7 case-patients were positive. We detected no SARS-CoV-2 RNA in any of the 62 urine specimens collected.

#### Specimens with Positive Results According to the SARS-CoV-2 rRT-PCR Assay

**Figure 2:**
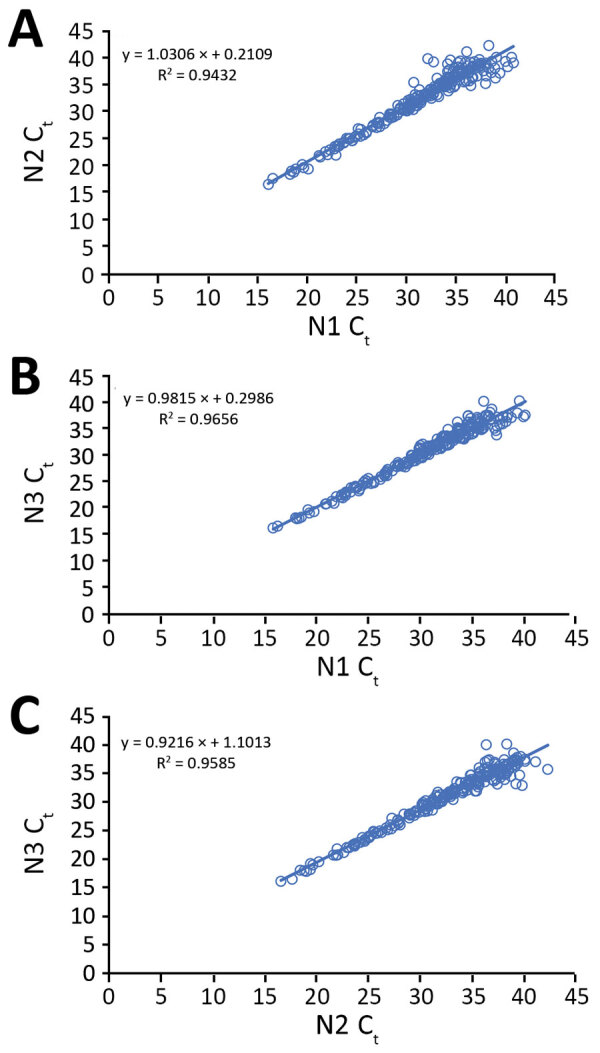
Comparison of the N1, N2, and N3 assays in the US Centers for Disease Control and Prevention real-time reverse transcription PCR panel for detection of SARS-CoV-2 with 223 SARS-CoV-2–positive clinical specimens. Linear regression lines were fitted to C_t_ values, with regression equations and coefficients of determination (R^2^). A) N1 vs. N2; B) N1 vs. N3; C) N2 vs. N3. C_t_, cycle threshold; SARS-CoV-2, severe acute respiratory syndrome coronavirus 2.

Of the 223 clinical specimens with positive results by all 3 rRT-PCR assays, C_t_ values obtained by the N1, N2, and N3 assays correlated well with each other (N1 vs. N2, R^2^ = 0.94; N1 vs. N3, R^2^ = 0.97; N2 vs. N3, R^2^ = 0.96) (Figure 2). Among the 71 pairs of nasopharyngeal and oropharyngeal specimens collected simultaneously from the 46 persons with suspected cases or from persons with laboratory-confirmed COVID-19 and any positive nasopharyngeal/oropharyngeal specimen, both nasopharyngeal and oropharyngeal samples were positive for 31 (43.67%); nasopharyngeal was positive but oropharyngeal was negative for 24 (33.80%); oropharyngeal was positive but nasopharyngeal was negative for 7 (9.86%); nasopharyngeal was positive but oropharyngeal was inconclusive for 5 (7.04%); and oropharyngeal was positive but nasopharyngeal was inconclusive for 4 (5.63%).

#### Inconclusive SARS-CoV-2 rRT-PCR Results

Inconclusive results were obtained for 40 (1.37%) of 2,923 specimens tested, including 1 (0.04%) of 2,437, from a person with a suspected case at initial testing, and 39 (8.02%) of 486 specimens collected for follow-up investigation from persons with laboratory-confirmed COVID-19. All specimens with inconclusive results had C_t_ values >35 (Table 9). Of these, 35 (87.5%) specimens were collected >7 days after symptom onset. Times from symptom onset to collection were unknown for the other 5 (12.5%) specimens.

**Table 9:** Inconclusive test results for human specimens with the US CDC real-time RT-PCR panel for detection of SARS-CoV-2*

Specimen ID	Initial test, C_t_				Retest, C_t_			Days after onset
	N1	N2	N3		N1	N2	N3	
Specimen from suspected cases								
NP1	Neg	39.3	Neg		Neg	Neg	36.9	Unknown†
Serial follow-up specimens from laboratory-confirmed COVID-19 cases								
NP1	36.1	39.8	Neg		Neg	Neg	37.5	15
NP2	39.6	39.6	Neg		36.9	38.5	Neg	16
NP3	41.6	Neg	Neg		37.8	Neg	39.4	12
NP4	38.1	Neg	Neg		37.0	38.5	Neg	14
NP5	Neg	37.8	36.1		Neg	38.3	36.8	16
NP6	Neg	40.1	Neg		Neg	37.5	37.0	22
NP7	Neg	38.3	36.1		37.2	Neg	Neg	13
NP8	38.3	Neg	36.0		36.7	38.1	Neg	22
NP9	Neg	39.9	Neg		Neg	38.7	Neg	25
NP10	Neg	Neg	36.4		Neg	Neg	37.6	11
NP11	36.6	Neg	36.2		Neg	38.0	Neg	13
NP12	37.5	39.2	Neg		Neg	38.9	36.0	15
NP13	36.9	Neg	39.5		Neg	36.9	37.0	13
NP14	36.4	40.2	Neg		36.2	Neg	Neg	17
NP15	37.0	Neg	Neg		Neg	37.2	Neg	18
NP16	Neg	39.1	35.8		38.0	37.3	Neg	11
NP17	35.4	37.9	Neg		Neg	38.7	36.9	16
NP18	Neg	37.8	36.1		36.7	37.2	Neg	Unknown‡
NP19	35.5	39.0	Neg		39.8	Neg	Neg	Unknown‡
OP1	Neg	38.0	Neg		35.9	Neg	Neg	18
OP2	36.3	38.2	Neg		38.4	Neg	Neg	20
OP3	Neg	39.1	Neg		Neg	38.4	37.8	11
OP4	Neg	38.2	37.3		35.8	Neg	36.0	10
OP5	Neg	37.2	36.7		37.7	Neg	Neg	7
OP6	Neg	Neg	36.5		38.1	Neg	Neg	9
OP7	Neg	Neg	38.4		Neg	Neg	36.6	9
OP8	37.2	38.2	Neg		37.2	Neg	36.8	11
OP9	Neg	37.6	38.1		Neg	Neg	39.3	15
OP10	36.6	38.0	Neg		Neg	39.3	Neg	9
OP11	Neg	Neg	Neg		Neg	Neg	Neg	Unknown‡
OP12	Neg	37.8	Neg		37.6	Neg	37.6	Unknown‡
Sputum 1	Neg	42.9	37.3		Neg	Neg	38.6	10
Sputum 2	38.0	Neg	Neg		36.1	38.2	Neg	12
Serum 1	39.8	Neg	Neg		Neg	39.6	Neg	6
Serum 2	38.1	Neg	Neg		Neg	Neg	35.8	16
Stool 1	Neg	Neg	38.3		Neg	40.1	Neg	21
Stool 2	Neg	38.8	Neg		36.9	39.2	Neg	13
Stool 3	36.0	Neg	Neg		37.3	Neg	Neg	15
Stool 4	Neg	39.9	Neg		37.7	Neg	35.8	9

*CDC, Centers for Disease Control and Prevention; C_t_, threshold cycle; COVID-19, coronavirus disease; N, nucleocapsid protein gene; neg, negative; NP, nasopharyngeal; OP, oropharyngeal; rRT-PCR, real-time reverse transcription PCR; SARS-CoV-2, severe acute respiratory coronavirus 2. †Repatriated from Diamond Princess Cruise Ship, Japan. ‡Repatriated from Diamond Princess Cruise Ship and confirmed positive in Japan.

#### Quality Control Data

Among 185 no-template controls and 219 human specimen controls included with specimen testing, negative results were obtained for all (100%) controls for N1, N2, and N3 assays and expected RP values were observed for human specimen controls. C_t_ values obtained from positive template control of 185 runs were in the expected range (data not shown).

## Discussion

The COVID-19 pandemic has affected multiple countries, causing severe illness and death, with sustained and efficient person-to-person community transmission, and it continues to pose a serious public health threat. Rapid and reliable laboratory diagnosis of SARS-CoV-2 infection is a critical component of public health interventions to mitigate this threat.

Our assay design and validation strategy were guided by several principles. First, we based assay designs on previous diagnostic assays that had been developed for detection of MERS-CoV (*12*) and SARS-CoV (*10*) and targeted the N gene. Because of the relative abundance of N gene subgenomic mRNA produced during virus replication (*13*), rRT-PCR assays targeting the N gene of coronaviruses could theoretically achieve enhanced diagnostic sensitivity. One study also showed that the N gene–based rRT-PCR assay was more sensitive than the open reading frame (ORF) 1 assay for detection of SARS-CoV-2 in clinical specimens (*14*). Second, we designed rRT-PCR assays on the basis of limited genetic information available soon after the emergence of SARS-CoV-2, when it was announced that a novel coronavirus of zoonotic origin was described as being similar to bat-SARS–like CoVs and only 1 SARS-CoV-2 sequence was publicly available. The N3 assay was intentionally designed to universally detect SARS-CoV-2 and other SARS-like sarbecoviruses to ensure detection of SARS-CoV-2 as this virus evolves over time and to improve early identification of future emerging novel coronaviruses from this high-risk subgenus. After completion of this study, the sequence of a new bat-SARS–like CoV, RaTG13 (EPI_SL_402131), was released on GISAID. Detected in 2013 from China, this virus appears to be the nearest bat precursor of SARS-CoV-2 (*15*), having 96% genome and 97% N gene sequence identity with SARS-CoV-2. All 3 assays are predicted to detect the RaTG13 strain. Third, we designed and used multiple assays for routine specimen screening to confirm virus detection when present at low concentrations and to reduce the possibility of false-negative results caused by polymorphisms within the binding sites of the target sequences, which might occur as the virus evolves. Fourth, we validated all assays by using multiple specimen types, including upper and lower respiratory specimens, serum, and stool samples, all of which have been shown to be diagnostically valuable for detection of SARS-CoV and MERS-CoV (*10*,*16*).

A study of SARS-CoV-2 viral load in upper respiratory tract specimens of infected patients showed that viral loads were higher in the nasopharynx than in the throat (*17*). Our study also showed a higher detection rate for nasopharyngeal swab samples than for oropharyngeal swab samples, although in some cases, viral load was higher in oropharyngeal than in nasopharyngeal swab samples. Our limited sputum data showed that SARS-CoV-2 seems to be more often detected and with higher viral loads in lower respiratory tract specimens than in upper respiratory tract specimens, especially later in the course of infection (72.22% positivity rate for sputum compared with 46.51% for nasopharyngeal and 30.00% for oropharyngeal samples in serial follow-up specimens). This phenomenon could be explained by the active replication of SARS-CoV-2 in the lungs, where the SARS-CoV-2 angiotensin–converting enzyme 2 receptor predominates (*18*). Similar to findings for SARS-CoV (*19*), our results showed that the SARS-CoV-2 RNA detection rate was high in the serial follow-up stool specimens collected from case-patients with laboratory-confirmed COVID-19. In contrast, SARS-CoV and MERS-CoV could be detected in serum/blood during the early prodromal phase of infection (*12*,*20*), whereas SARS-CoV-2 RNA was not detected in any of the serum specimens during the initial testing, although it was detected in serum collected from 2 (13.3%) case-patients >9 days after symptom onset. Similarly, whereas SARS-CoV and MERS-CoV RNA have been detected in urine (*16*,*19*), SARS-CoV-2 RNA was not detected in urine in our study nor has detection been reported (*21*).

All specimens with inconclusive results in our study had C_t_ values >35, with reactivity not attributable to 1 individual assay, indicating that the viral RNA levels in the specimens were at the LoD of the assay. All specimens with inconclusive results were collected either >7 days after symptom onset or from repatriates who had been quarantined on the Diamond Princess cruise ship for ≈2 weeks before specimen collection. Inconclusive results most likely resulted from low viral loads, especially in upper respiratory tract specimens collected later in the course of infection. This observation is supported by another study, which showed that SARS-CoV-2 actively replicated in the oropharynx during the first 5 days after symptoms onset only (*21*). If inconclusive results are obtained, collection of additional specimens and specimen types may be warranted for accurate diagnosis.

All 3 SARS-CoV-2 rRT-PCR assays proved to be both sensitive and specific with high reproducibility. The earliest specimens from US COVID-19 case-patients were confirmed by virus isolation, whole-genome or partial gene sequencing, or both (*22*) (GenBank accession nos. MN985325, MN988713, MN994467–8, MN997409, MT027062–4, MT039887–8, MT044257–8, MT106052–4, MT118835, MT159705–22, and MT184907–13), although not all positive follow-up specimens detected by rRT-PCR assays were confirmed by independent assays. An independent comparison study showed that the CDC N2 and N3 assays performed well among 7 assays targeting the N gene that were evaluated and posted by the World Health Organization (Y. Jung, unpub. data, https://www.biorxiv.org/content/10.1101/2020.02.25.964775v1). In another study that compared 7 assays, the N2 assay was shown to be among the most sensitive (*23*). However, both studies used enzyme chemistries not optimized by CDC for testing, and we did not observe difference in sensitivity among the 3 assays in our study.

To expedite reagent kit manufacturing by removal of the N3 assay from the panel, we analyzed results of 2,437 specimens for initial COVID-19 testing when only N1 and N2 assay results were used for interpretation. Positive and negative test results showed 100% agreement to interpretation with all 3 assays. Only 1 (0.04%) specimen with an inconclusive result, which was positive for N2 at initial testing and positive for N3 at retesting, would be reported as negative if the N3 assay was excluded from result interpretation of the CDC panel. This analysis demonstrates that interpretation of only N1 and N2 assay results agreed totally (99.96%) with the original results, and removal of the N3 assay from the panel would have a negligible effect on the ability of the test to detect positive specimens. The analytical LoD of the panel remained the same for detection of SARS-CoV-2 with or without the N3 assay included, and sensitivity of the panel was not affected. The benefits of testing with only N1 and N2 assays include simplified diagnosis of COVID-19 with fewer reactions for each patient specimen as well as increased testing throughput and reduced reagent cost, although removal of the N3 assay from the panel limits the ability to detect other SARS-like coronaviruses. Accordingly, the N3 assay has been removed from CDC testing interpretation, and current recommendations are to test with the N1 and N2 assays only (*11*). Because SARS-CoV-2 is an RNA virus with an estimated evolutionary rate of ≈1.8 × 10^−3^ substitutions/site/year (*24*), performance of the assays included in the CDC panel will be monitored as SARS-CoV-2 continues to circulate and evolve over time. Therefore, assay designs included in the CDC panel are subject to change to account for future genetic mutations in the SARS-CoV-2 genome that may affect test sensitivity.

In conclusion, the CDC rRT-PCR panel for detection of SARS-CoV-2 demonstrated high sensitivity and specificity for detecting 5 RNA copies/reaction with no observed false-positive reactivity, and it facilitated rapid detection of SARS-CoV-2 infections in humans. These assays have proven to be valuable for rapid laboratory diagnosis and support, clinical management, and infection prevention and control of COVID-19.
